# Cardiac disease diagnosis based on GAN in case of missing data

**DOI:** 10.1371/journal.pone.0292480

**Published:** 2024-11-22

**Authors:** Xing Chen, Na Zhang, Xiaohui Yang, Chunyan Wang, Qi Na, Tianyun Luan, Wendi Zhu, Chenjie Zhang, Chao Yang

**Affiliations:** 1 Jilin Province FAW General Hospital, Changchun, Jilin, China; 2 Changchun Guanghua University, Changchun, Jilin, China; 3 Changchun University of Science and Technology, Changchun, Jilin, China; 4 Beijing Aerospace Times Laser Inertial Technology Company, Ltd, Beijing, China; PLOS: Public Library of Science, UNITED STATES OF AMERICA

## Abstract

In daily life, two common algorithms are used for collecting medical disease data: data integration of medical institutions and questionnaires. However, these statistical methods require collecting data from the entire research area, which consumes a significant amount of manpower and material resources. Additionally, data integration is difficult and poses privacy protection challenges, resulting in a large number of missing data in the dataset. The presence of incomplete data significantly reduces the quality of the published data, hindering the timely analysis of data and the generation of reliable knowledge by epidemiologists, public health authorities, and researchers. Consequently, this affects the downstream tasks that rely on this data. To address the issue of discrete missing data in cardiac disease, this paper proposes the AGAN (Attribute Generative Adversarial Nets) architecture for missing data filling, based on generative adversarial networks. This algorithm takes advantage of the strong learning ability of generative adversarial networks. Given the ambiguous meaning of filling data in other network structures, the attribute matrix is designed to directly convert it into the corresponding data type, making the actual meaning of the filling data more evident. Furthermore, the distribution deviation between the generated data and the real data is integrated into the loss function of the generative adversarial networks, improving their training stability and ensuring consistency between the generated data and the real data distribution. This approach establishes the missing data filling mechanism based on the generative adversarial networks, which ensures the rationality of the data distribution while filling the missing data samples. The experimental results demonstrate that compared to other filling algorithms, the data matrix filled by the proposed algorithm in this paper has more evident practical significance, fewer errors, and higher accuracy in downstream classification prediction.

## Introduction

### Background

Cardiac disease is a major global health problem that affects millions of people every year. With the increasing prevalence of cardiac disease, early detection and accurate prediction of its occurrence are crucial for effective management and treatment. Advances in computer science and technology have provided new opportunities for the medical field to address this problem. Machine learning and artificial intelligence algorithms have been applied to analyze large datasets of medical information to develop predictive models for cardiac disease [1]. According to data from the United States Centers for Disease Control and Prevention (CDC), cardiac disease remains a leading cause of death for most races in the United States, including African Americans, American Indians, Alaska Native Americans, and whites [2]. Approximately 47% of Americans have at least three of the key risk factors for cardiac disease, such as high blood pressure, high cholesterol, and smoking, among others. Other important indicators include diabetes status, obesity (measured by high BMI), physical inactivity, and excessive drinking [3]. In China, the Interpretation of Key Points of China’s Cardiovascular Health and Disease Report 2021, released in 2021, clearly points out that the number of people suffering from cardiovascular diseases is on the rise, with rates of 45.91% in rural areas and 43.56% in urban areas. Early diagnosis and timely treatment are essential measures to address this crisis. Moreover, the incidence of cardiac disease in cardiovascular disease centers is increasing and gradually showing a younger trend, which has raised health alarms for more young people [4]. The economic burden of cardiovascular diseases on residents and society is also increasing, making it a major public health problem. Therefore, it is crucial to strengthen government-led prevention and treatment of cardiovascular diseases [5]. Cardiovascular disease is associated with high disability and mortality rates. Despite the advanced treatment methods available at present, many patients remain unable to care for themselves after being cured. As such, primary prevention of cardiovascular disease is particularly important. Primary prevention involves modifying unhealthy living habits before the onset of the disease, such as quitting smoking, adopting a balanced diet, increasing physical activity, and taking medications to control risk factors such as blood pressure, cholesterol levels, and blood sugar. Although the pathogenesis of cardiovascular diseases has not been fully determined, a significant number of studies have confirmed that all types of cardiovascular diseases share a common pathological basis, and the primary risk factors have been identified. Most of these factors can be managed through lifestyle modifications. Therefore, it is crucial to develop accurate and efficient early prediction tools for cardiovascular diseases to identify high-risk groups and provide early warnings. Furthermore, it is recommended that high-risk individuals change their unhealthy living habits to control risk factors and reduce the risk of developing cardiovascular disease [6]. However, at this stage, many rare diseases have limited data samples. Typically, people only seek medical attention when they experience discomfort. Take cancer, for example; by the time people experience discomfort, the tumor has often formed and may have already progressed to the middle or late stage of the disease. Before targeted drugs corresponding to cancer become available, doctors can only examine the patient’s DNA data and cluster them based on the detected data, assuming that the number of clusters is known in advance. Once the clustering results are obtained, different treatment methods are implemented based on the clustering results, as medical resources are valuable. However, the cost of DNA testing is high, and the number of patients with certain types of cancer is relatively small, making diagnosis more challenging.

### Related work

Traditional algorithms for processing missing data are mainly divided into two categories: deletion and filling. The deletion method involves obtaining a “complete” data set by directly deleting missing items. Its advantage is that it is simple and easy to operate; however, this method leads to information loss and deviation in data distribution, rendering it only applicable to cases where there is a small proportion of missing items. The filling method, on the other hand, can be divided into statistics-based filling and model-based filling. The former includes mean filling, random filling, forward (backward) interpolation, weighted filling, and other methods that utilize the statistical characteristics of the missing data to fill in the gaps. These algorithms yield stable filling results and are applicable to situations where the distribution characteristics of the missing sequence are simple and clear, and the variable correlation is strong. The model-based filling methods, such as regression model filling, K-nearest neighbor interpolation, and the expectation maximization algorithm (EM) filling, involve selecting different models or algorithms to measure the distance between missing and observed data. The goal is to adjust the parameters to minimize this deviation and estimate missing data. This approach makes full use of the information contained in the existing data, but has specific requirements for the data set itself, such as strong correlations and a specific distribution of the data. Overall, there are various algorithms for processing missing data, each with its own advantages and limitations. The selection of an appropriate method depends on the characteristics of the data set and the specific research question.

Numerous algorithms have been proposed for filling missing data, with discriminant and generative algorithms being the two advanced approaches. Discriminant algorithms include chain equation multiple filling algorithm, random forest algorithm, and matrix filling algorithm, while generative algorithms include expectation maximization algorithms and deep learning-based algorithms, such as noise reduction automatic encoders (DAE) [7] and generating adversarial networks (GAN) [8]. However, some of these algorithms have limitations. For instance, Fedus [9] et al. generate timing data based on self-coder, but the training process of this algorithm requires complete timing data, which makes it difficult to guarantee the generation effect when the training data is missing. Gheyas [10] et al. solved the filling problem through denoising self-coder, but their algorithm uses the average value or the most commonly used label to replace the missing data when initializing the network, which may distort the multivariate relationship. Che [11] et al. developed the GRU-D model, which achieves better prediction results, but cannot be used for unsupervised learning without prediction tags. Yoon [12] et al. applied the generation countermeasure network to the generation of missing data but did not take into account the dependency between the time series data. Zheng [13] et al. proposed a fault prediction method based on double GAN, which is mainly aimed at the situation where the training samples are small but the data set is continuous and complete. Luo [14] et al. used GRU to form GAN to realize the generation of time series data, but this algorithm cannot guarantee that the filling result of missing data conforms to the overall distribution. Therefore, while various algorithms have been developed for filling missing data, each algorithm has its limitations and is best suited to certain types of data sets.

The EM algorithm for missing data imputation was first proposed in 1977 by Dempster, Laird, and Rubin, and has since attracted significant attention and research from scholars [15]. It is an iterative algorithm based on maximum likelihood estimation, which simplifies a complex estimation problem by assuming the existence of hidden variables and simplifying the likelihood equation. Through subsequent improvements and applications, such as the ECM algorithm [16] proposed by Meng and Rubin, and the MCEM algorithm [17] that combines Monte Carlo simulation with the E-step, the EM algorithm has become a widely used and stable parameter estimation method for missing data imputation. Recent research has expanded the methods for missing data imputation from simple methods like mean filling and Hotdecking to more complex model-based methods like regression filling, statistical learning methods such as missing forest filling and automatic encoding filling. These advancements have reduced the requirements for data hypothesis and improved the accuracy of missing data imputation.

It is true that there are not many technical means to fill the missing data based on deep learning, and the research on filling the missing data using Generative Adversarial Networks is still relatively new. However, GAN has shown great potential in generating realistic data and filling missing data [18]. GAN has been used for image generation and image reconstruction, and these tasks are similar to missing data filling, as they both involve learning the underlying distribution of data and generating data sets subject to sample distribution under certain constraints. Unlike traditional missing data filling methods that rely on strict assumptions, GAN does not have any explicit assumptions about data, and they can learn complex relationships and patterns in the data. The larger the amount of data, the more accurate the results will be, as GAN can use the additional data to improve the accuracy of the generated data. However, GAN can also face challenges such as mode collapse or gradient disappearance during the training process [19], which can affect the quality of the generated data.

### Our work

The algorithms mentioned above have yielded promising results in the context of data filling. However, certain issues still need to be addressed. Firstly, the error between the filled data and the real data is substantial, and the generator is unable to generate a sample distribution that closely matches the missing data. Secondly, the generator produces values between 0 and 1, rather than 0 or 1, which can make it challenging to interpret the actual meaning of the generated data. The main contributions of this paper are as follows:

1. We propose a new missing data filling architecture called AGAN, which is based on Generative Adversarial Networks. AGAN does not use prompt matrix but directly uses a similar discriminator network for filling, which effectively improves the filling accuracy.
2. We introduce the attribute matrix and directly convert it into real-values, two-values and nominal values. This effectively addresses the issue of the unclear interpretation of the generated data.
3. Our experimental results demonstrate that the proposed AGAN algorithm outperforms other mainstream filling algorithms in terms of lower error rates and higher downstream prediction accuracy.

## Dataset

This study utilizes two real heart disease datasets [20]. The Table 1 below provides an overview of the sample and variable characteristics of these two datasets:

**Table 1:** Overview of attribute information of real heart disease datasets.

Dataset	sample size	features	real-values	two-values	nominal values
UCI Heart	1025	14	6	4	4
Kaggle Heart	319795	18	5	11	2

**Fig 1:**
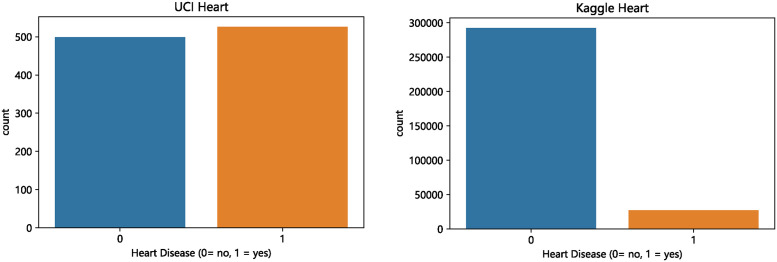
Data distribution of two real heart disease datasets.

The number of samples, attributes, real-values, two-values, and nominal values in these datasets differ. Conducting experiments using these data sets can reflect the performance differences of the missing data filling model in different dimensional data sets. In the case of the real heart disease data sets, it is necessary to extract the corresponding features and labels. For instance, in the Kaggle Heart dataset, heart disease is represented by “Yes”, and no disease is represented by “No”, so the corresponding “Yes” should be converted to 1, and “No” should be converted to 0. The data distribution of the two data sets is illustrated in Fig 1.

In this study, we used two main datasets: UCI Heart and Kaggle Heart. These two datasets significantly differ in the number of positive and negative data entries, which is crucial for understanding and assessing the characteristics of the datasets as well as the difficulty of the prediction tasks. In the UCI Heart dataset, the number of positive and negative data entries is balanced, providing us with a relatively balanced data environment to evaluate the model’s performance in dealing with balanced data. On the other hand, the Kaggle Heart dataset shows an imbalanced data distribution, where one category has significantly more entries than the other. This characteristic of category imbalance poses additional challenges for prediction tasks, especially in preventing model bias towards the majority class while ensuring sufficient recognition ability for the minority class. We have documented the category distribution in these two datasets in detail in our research to ensure that this key factor is adequately considered in subsequent analyses and model evaluations.

**Fig 2:**
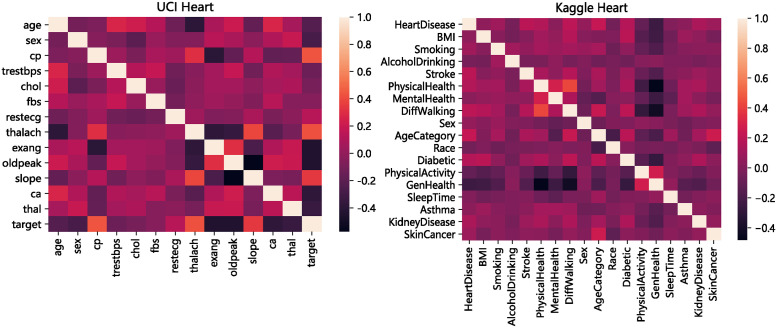
Correlation coefficient matrix of two heart disease datasets.

Since both datasets have a high number of features, we can analyze the correlation coefficient matrix of the two datasets to gain insight into their relationships, as shown in Fig 2. The graph provides a visual representation of the correlation between variables, with colors indicating the strength of the relationship. Darker colors indicate weaker correlations.

### UCI Heart dataset

The UCI Heart Disease Databases was compiled by Andras Janosi, William Steinbrunn, Matthias Pfisterer, and Robert Detrano. The dataset consists of 1025 samples, each of which has 14 attribute information.

### Kaggle Heart dataset

The Kaggle Heart dataset is sourced from the United States Centers for Disease Control and Prevention as a crucial part of the Behavioral Risk Factor Monitoring System (BRFSS), which conducts annual telephone surveys to gather health-related data of American residents. The BRFSS system undertakes over 400,000 adult interviews annually, making it the most comprehensive and continuous health survey system worldwide. This dataset includes 319,795 samples, each consisting of 18 attribute information, such as HeartDisease, BMI, Smoking, AlcoholDrinking, Stroke, PhysicalHealth, MentalHealth, DiffWalking, Sex, AgeCategory, Race, Diabetic, PhysicalActivity, GenHealth, SleepTime, Asthma, KidneyDisease, and SkinCacer.

## Methodology

### Model overview

**Fig 3:**
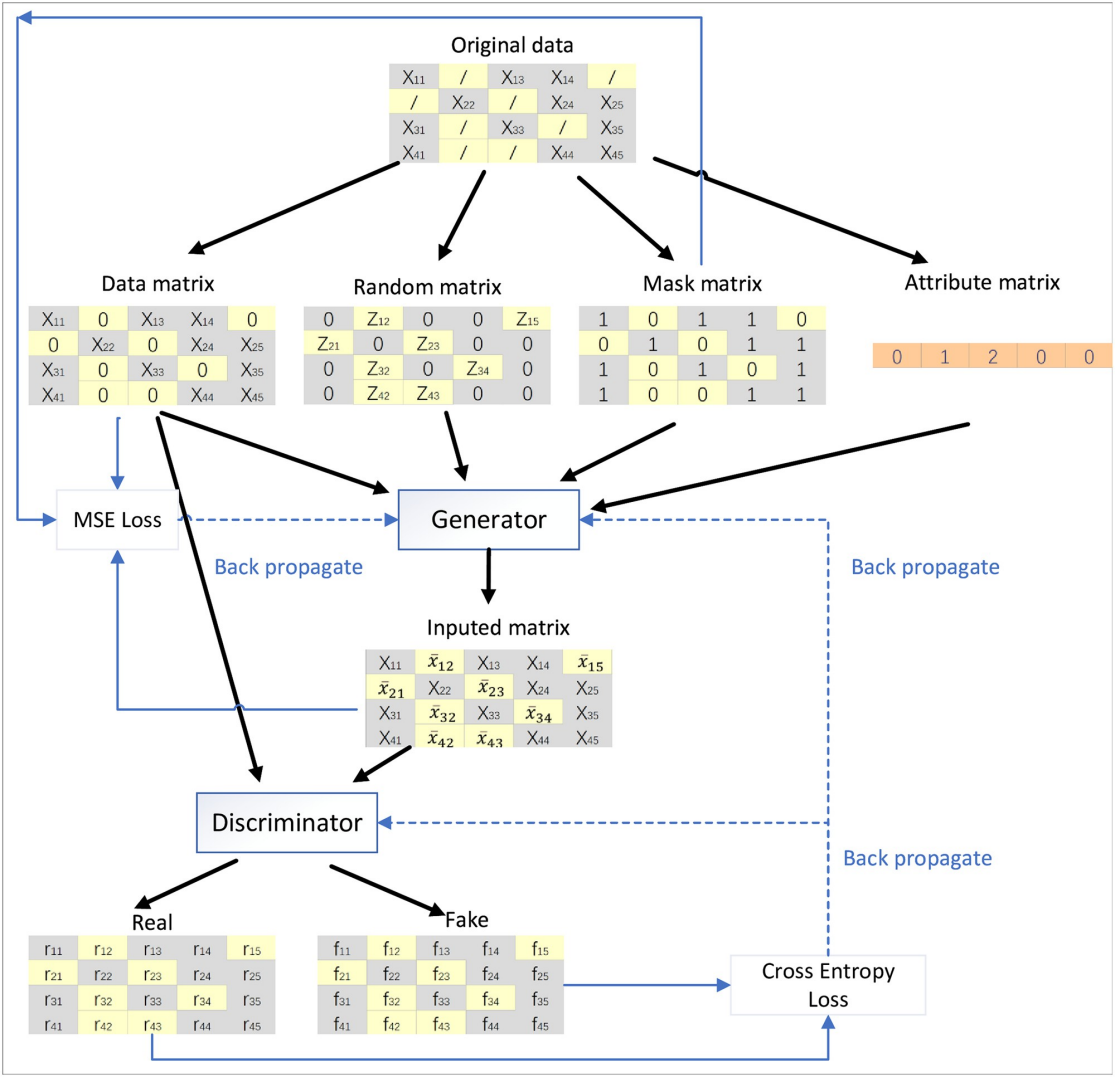
Original data example.

This paper proposes a new missing data filling architecture AGAN based on generative adversarial networks to address the issue of diverse feature attributes in medical data, specifically in heart disease datasets [21]. The proposed method generates a feature attribute matrix for a given dataset *X* = (*X*_1_, *X*_2_, …, *X*_*n*_)^*T*^ consisting of n data samples, each with d-dimensional attribute values *X*_*i*_ = {*x*_*i*1_, *x*_*i*2_, …, *x*_*id*_} For instance, missing data on a 4*5 scale as shown in Fig 3 can be represented as *X* = (*X*_1_, *X*_2_, *X*_3_, *X*_4_, *X*_5_)^*T*^ where “*x*_*i*__*j*_” represents observable data, and “/” represents missing data [22].

**Fig 4:**
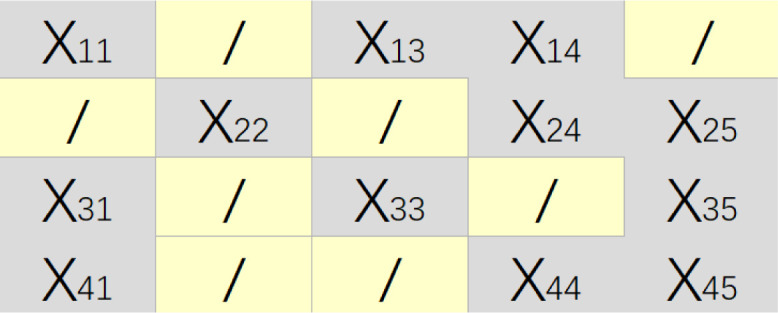
Mask data example.

Because the original data matrix X is incomplete, it is necessary to have another corresponding matrix to determine the information of the missing data [23]. The missing marker matrix *M* = {*m*|*m*_*ij*_ ∈ {0, 1}} is a binary matrix used to identify the location of the missing data, as shown in Fig 4. The elements in matrix M are generated by the following formula:

$$\begin{array}{c} m_{ij}=\{\begin{array}{c} 0,\begin{array}{cc} \; & if\;\begin{array}{cc} x_{ij}=NaN & \; \end{array} \end{array}\\ 1,\begin{array}{cc} \; & if\;\begin{array}{cc} x_{ij}\neq NaN & \; \end{array} \end{array} \end{array},1\leq i\leq n,1\leq j\leq d \end{array}$$ (1)

A value of 0 for *m*_*ij*_ ndicates that the jth attribute value of the ith sample is missing.

**Fig 5:**
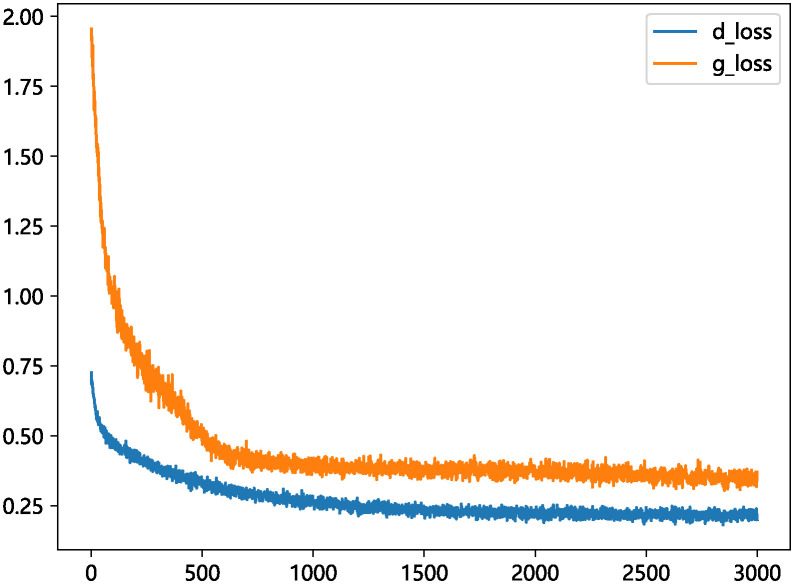
Attribute matrix example.

Feature attributes can be categorized into real-value type, two-value type, and nominal value type, where most of them are numerical data, while a few attributes, such as gender and whether or not, are Boolean values (0 or 1) and some are semantic discrete values such as pain type, rising and falling levels, etc. Hence, the real-value type, two-value type, and nominal value type can be represented by 0, 1, and 2, respectively [24]. Specifically, the attribute matrix is presented in Fig 5, where matrix *A* = {*a*|*a*_*i*_ ∈ {0, 1, 2}} is used to identify the attribute information of the data. The first, fourth, and fifth columns correspond to real value type, the second column corresponds to binary type, and the third column corresponds to nominal type [25].

$$\begin{array}{c} \begin{array}{c} a_{i}=\{\begin{array}{c} 0ifdtype(X_{i})=float\\ 1ifdtype\left(X_{i}\right)=\text{int}64\&shape\left(unique\left(X_{i}\right)\right)=2\\ 2else \end{array},\\ 1\leq i\leq n \end{array} \end{array}$$ (2)

The addition of the attribute matrix provides additional information, which can constrain sample generation to a range closer to real samples and fill in more realistic data.

**Fig 6:**
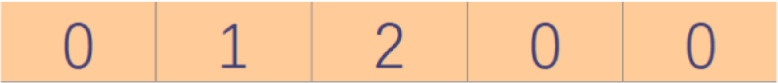
Architecture of AGAN algorithm model.

The overall model architecture of AGAN is shown in Fig 6.

### Generator

The role of the generation network (represented as G) in the generative adversarial network model is to learn to fit the data distribution and generate new data. In the context of missing data filling, generating data at the missing locations is a key task. Therefore, the function of the generation network in the data filling task is to generate data. The network is generated by first fitting the distribution of real data and, in this process, learning the mapping relationship between random noise data and real data. Within the generation network, the input data includes the data matrix X, random noise data Z, marking matrix M, and attribute matrix A. The generated data is expressed in matrix $\bar{X}$. The complete data, expressed as matrix $\hat{X}$, is obtained by combining the generated data obtained through the generation network with the original data that is not missing. Z is a d-dimensional vector, represented as *Z* = (*Z*_1_, *Z*_2_, …, *Z*_*d*_), where each element of ${\left\{Z_{i}\right\}}_{d}^{i=1}$ has a non-missing value in ${\left\{X_{i}\right\}}_{d}^{i=1}$, and the missing values in ${\left\{X_{i}\right\}}_{d}^{i=1}$ are replaced by random noise. The attribute matrix *N* = (*N*_1_, *N*_2_, …, *N*_*d*_) marks the attribute information of each column in the dataset. The function that extracts random values from a continuous uniform distribution (i.e. [-0.01, +0.01]) outputs:

$$\begin{array}{c} Z=M⊙X⊕(1-M)⊙N \end{array}$$ (3)

$\bar{X}$ is the fill value generated by the generator, and its formula is as follows:

$$\begin{array}{c} \bar{X}=G\left(X,Z,M,A\right) \end{array}$$ (4)

$\hat{X}$ represents the final complete data. In cases where there is a missing value, the corresponding data in X is used. Conversely, where there are no missing values, the data in $\hat{X}$ is used. Attribute matrix A is used to mark the relevant attributes. If the data is binary or nominal, it will be converted to the nearest integer value. Otherwise, the data will remain unchanged. The overall formula is as follows:

$$\begin{array}{c} \hat{X}=A\left(M⊙\bar{X}+\left(1-M\right)⊙X\right) \end{array}$$ (5)

### Discriminator

In generative adversarial networks, the discriminator network (represented as D) engages in an antagonistic game with the generator network [26]. Its main function is to determine whether the input is from real data or data generated by the generator network, and to score the input data and provide feedback to the generator network. This process is crucial in achieving the most desirable convergence state through antagonistic training, and embodies the idea of a zero-sum game.

In the missing data filling task, the function of the network is evaluated based on the data generated by the generator network. The input data is distinguished as either derived from real data or generated data from the generator network, and the differentiation result is fed back to the generator network. Consequently, the generator network adjusts its performance based on the feedback provided by the discriminator network, thereby generating data that more closely resembles real data [27].

### Data preprocessing and model training

The first step in the missing data filling task is data preprocessing [28]. The objects of this task may include real-values, two-values, and nominal values, which cannot be directly used as input data for the filling model. Additionally, the NaN marks in incomplete data cannot be calculated. Therefore, data preprocessing is necessary for data filling. In the key steps of data preprocessing, we first employed One-Hot Encoding for categorical data, converting non-numeric data into a numeric format to meet the requirements of the model. Then, for numerical data, we performed standardization to ensure all feature values are on the same scale, reducing the impact of differences in feature value ranges on model predictive performance. Additionally, we split the dataset into training and test sets, which not only helps in assessing the model’s generalization ability but also provides a suitable data environment for subsequent model training and testing. Finally, we conducted detailed feature engineering, including selecting the most meaningful features for the prediction task and performing feature construction or transformation as needed by the model. This step aims to further enhance the model’s performance and accuracy. These comprehensive data preprocessing measures provide an accurate and reliable data foundation for our research, ensuring the effectiveness of model training and evaluation.

To prevent possible problems of gradient disappearance and gradient explosion in model training, and to enable the loss function in training to converge quickly, data normalization is performed [29]. The normalization method used in this paper is maximum and minimum normalization, which does not affect the properties of categories 0 and 1. The normalization formula is as follows:

$$\begin{array}{c} x_{scale}=\frac{x_{ij}-\text{min}\left(d_{j}\right)}{\text{max}\left(d_{j}\right)-\text{min}\left(d_{j}\right)} \end{array}$$ (6)

Where max(*d*_*j*_) and min(*d*_*j*_) represent the maximum and minimum values of the j-th dimension attribute, and the normalized data value *x*_*scale*_ is within the range of [0, 1].

In order to solve the common problems of gradient disappearance and gradient descent in the networks, this paper introduces the variable fraction gradient descent method. There are three definitions of fractional derivatives: Riemann-Liouville, Caputo, and Grunwald-Letnikov fractional derivatives. Caputo fractional derivative, which is very suitable for engineering calculation, is used in this paper, and its definition is as follows:

acDtμf(t)=1Γ(n-μ)∫at(t-τ)n-μ-1f(n)(τ)dτ,(n-1≤μ<n,t>a) (7)

Compared to traditional methods, the iteration of the variable fraction gradient descent method can locate a better global minimum more efficiently. This translates to faster training speed and improved training accuracy compared to the traditional neural network training approach. The generator G and discriminator D typically utilize the ReLU or sigmoid function as the network activation function. However, with the ReLU function, model collapse may occur during network parameter updates, which results in neuron disappearance and the gradient flow being disrupted when the descending gradient is large. To address these issues, this section introduces a new function to modify the linear element, utilizing variable fractional order, fractional gradient descent, and gradient ascent methods in place of the traditional gradient descent or ascent methods. An alternative activation function, the Swish activation function, proposed by Google, offers improved effectiveness over ReLU [30]. Swish boasts the advantages of a lower bound, monotony, and differentiability throughout. Moreover, Swish introduces adaptive parameters that adjust the negative slope based on specific conditions, thus enhancing activation efficiency. It is defined as:

$$\begin{array}{c} Swish\left(x\right)=x\cdot sigmoid\left(\beta x\right)=\frac{x}{1+e^{-\beta x}} \end{array}$$ (8)

Where *β* represents the adaptive parameter. As the positive half-axis of the Swish function is linear, the curves of the first and second derivatives are smooth, making gradient calculation straightforward. Consequently, the operational cost of the neural network is reduced, and its operational efficiency is improved.

During network training, the generator and discriminator are cross-trained, with the discriminator trained k times every time the generator is trained in a confrontation [31]. To train the generator, the discriminator parameters are fixed and connected in series to the generator. The prediction loss of the generator is then obtained from the difference between the filled data generated by the generator and the real data. The output probability value of the discriminator is used to calculate the confrontation loss of the generator, and the gradient information of the generator network parameters is computed by the loss function. The parameters are then updated along the negative gradient direction. When training the discriminator, the parameters of the generator are fixed, and false filling data are generated. The discriminator judges and outputs the probability that the current input data originates from the real sample set. The overall network training algorithm is presented in Table 2.

**Table 2:** Training algorithm flow.

AGAN algorithm
Input:raw data with missing data X; batch mb; generator loss
hyperparameter *α*; discriminator loss hyperparameter λ;
Iterations n_iter; The number of times the generator
trains the discriminator per training k;
for iter ← 1 to n_iter do
for critic ← 1 to n_critic do
raw data with missing data X: ${\left\{\tilde{x}\left(j\right)\right\}}_{j=1}^{mb}$
mask data M: ${\left\{\tilde{m}\left(j\right)\right\}}_{j=1}^{mb}$
random noise N:${\left\{\tilde{n}\left(j\right)\right\}}_{j=1}^{mb}$
attribute data A:$A:{\left\{\tilde{a}\left(j\right)\right\}}_{j=1}^{mb}$
for j ← 1 to mb do
$\tilde{z}\left(j\right)\leftarrow \tilde{m}\left(j\right)⊙\tilde{x}\left(j\right)⊕\left(1-\tilde{m}\left(j\right)\right)⊙\tilde{n}\left(j\right)$
$\bar{x}\left(j\right)\leftarrow G\left(\tilde{z}\left(j\right),\tilde{m}\left(j\right),\tilde{a}\left(j\right)\right)$
$\hat{x}\left(j\right)\leftarrow \tilde{a}\left(j\right)\left(\tilde{m}\left(j\right)⊙\bar{x}\left(j\right)+\left(1-\tilde{m}\left(j\right)\right)⊙\tilde{x}\left(j\right)\right)$
end for
Update discriminator D with Adam optimizer
$\nabla_{D}\frac{1}{mb}\sum_{j=1}^{mb}L_{D}\left(D\left(\tilde{x}\left(j\right)\right),D\left(\bar{x}\left(j\right)\right),\tilde{m}\left(j\right)\right)+\frac{\lambda }{mb}\sum_{j=1}^{mb}L_{GradPenalty}\left(D\left(\hat{x}\left(j\right)\right)\right)$
end for
Update generator G with Adam optimizer
$\nabla_{G}-\frac{1}{mb}\sum_{j=1}^{mb}L_{G}\left(D\left(\bar{x}\left(j\right),\tilde{m}\left(j\right)\right)\right)+\frac{\alpha }{mb}\sum_{j=1}^{mb}L_{MSE}\left(\tilde{x}\left(j\right),\bar{x}\left(j\right),\tilde{m}\left(j\right)\right)$
end for
*Z* ← *M* ⊙ *X* ⊕ (1 − *M*) ⊙ *N*
$\bar{X}\leftarrow G\left(Z,M,A\right)$
$\hat{X}\leftarrow A\left(M⊙X⊕\left(1-M\right)⊙\bar{X}\right)$
Output:$\hat{X}$

## Experiments & discussion

### Experimental environment

The hardware and software environment utilized in this experiment is shown in Table 3.

**Table 3:** Experimental hardware and software environment.

environment	value
CPU	Intel(R) Core(TM) i9-10920X CPU@3.50GHz
harddisk	1T SSD
RAM	128GB
OS	Windows 10
program language	Python 3.7
deep learning framework	Pytorch 1.10
GPU	NVIDIA GeForce RTX3090

### Performance metrics

1. Root mean square error (RMSE)
  The root mean square error (RMSE) is the evaluation index used in this paper to assess the experimental results. RMSE measures the difference between data and is utilized in this experiment to calculate the error between the real and filled data. The calculation formula for RMSE is as follows:
  $$\begin{array}{c} \begin{array}{cc} & RMSE\\ & =\sqrt{\frac{1}{\sum \left(1-m_{ij}\right)}\times \sum_{ij}{\left(\left(1-m_{ij}\right)\times x_{ij}-\left(1-m_{ij}\right)\times {\hat{x}}_{ij}\right)}^{2}} \end{array} \end{array}$$ (9)
  The RMSE utilized to evaluate the filling effect in this experiment only calculates the error between the missing value and its corresponding filling value, and does not factor in the observed data in the data sample during the calculation. This approach helps avoid the impact of different missing rates on the filling evaluation. The calculation of RMSE is based on the normalization of the original data, a step aimed at eliminating the impacts of different dimensions and ranges of values. Subsequently, these normalized data are used to train the AGAN model and for the imputation of missing values. The specific method of calculating RMSE involves comparing the differences between the normalized real data and the predicted data from the model. This entails computing the squared differences between the predicted and actual values for each data point, then averaging these differences across all data points, and finally taking the square root of this average to obtain the RMSE value. In determining the nominal value of RMSE, the focus is primarily on the statistical differences between the model’s predicted values and the actual values. Since the study concentrates on the imputation of missing values in continuous data, RMSE in this context is mainly used to assess the prediction accuracy of these continuous data.
2. Proportion of falsely classified (PFC)
  The second evaluation index used in this paper is PFC, which is the performance index for category variables. A smaller value and a smaller error indicate a better filling effect. The calculation formula for PFC is as follows:
  $$\begin{array}{c} PFC=\frac{\sum_{ij}I(X_{\left(i,j\right)}^{true}\neq X_{\left(i,j\right)}^{imp})}{N_{A}} \end{array}$$ (10)
  In the above equation,$X_{\left(i,j\right)}^{true}$ represents the value of the j-th variable of the i-th sample in the complete dataset, $X_{\left(i,j\right)}^{imp}$ represents the value of the j-th variable of the i-th sample in the filled dataset, and *N*_*A*_ is the number of missing discrete variables.
3. Area Under Curve and receiver operating characteristic curve
  This paper utilizes the AUC (Area Under Curve) index to reflect the indirect comparison between the two classification models used in the experiment. AUC represents the area under the subject’s operating curve. The AUC index can assess the quality of the classifier even when the sample label distribution is uneven. The calculation of AUC is dependent on the number of positive true positive rates (TPR) and false positive rates (FPR). The calculation formula for FPR and TPR is shown below:
  $$\begin{array}{c} TPR=\frac{TP}{TP+FN} \end{array}$$ (11)
  $$\begin{array}{c} FPR=\frac{FP}{FP+TN} \end{array}$$ (12)
  By plotting FPR as the ordinate and TPR as the abscissa, the receiver operating characteristic curve can be generated. AUC is the area under this curve, i.e.:
  $$\begin{array}{c} AUC=\int_{-\infty }^{\infty }y(t)dx(t) \end{array}$$ (13)
  Where x and y are the horizontal and vertical coordinates of the curve in the figure.
  AUC represents the probability of a classifier ranking a randomly chosen positive sample higher than a randomly chosen negative sample. In a binary classification model, a higher AUC indicates that the classifier has better discrimination power between positive and negative samples. The AUC index is not affected by the distribution proportion of positive and negative samples, making it a useful metric in situations where the sample size or class distribution is imbalanced. Compared to metrics such as recall and accuracy, AUC can more accurately assess the quality of a classification model in such situations.

### Experimental results and discussion

In our study, the training parameters of the AGAN model were carefully chosen to ensure optimal performance and effective convergence. We used a learning rate of 1e-6, a decision based on preliminary experimental results, aimed at ensuring stable convergence of the model while avoiding problems associated with too rapid convergence. To balance computational resources and model performance, we set the batch size to 128. Additionally, the length of the generator was set to 96, which helps the model generate more accurate and meaningful data sequences. Before formal training, we pre-trained the model for 30 epochs to enhance its stability and predictive performance. The total training process of the model included 200 training epochs to ensure the model thoroughly learns the data features. In our model, the number of hidden units was set to 64, providing sufficient model complexity. Also, we used a 64-dimensional noise vector as input to the generator to enhance the diversity of the data. Finally, to balance the training between the generator and discriminator, we set the discriminator to update once for every 8 iterations of the generator. These parameter settings were based on an in-depth understanding of the dataset characteristics and the results of a series of preliminary experiments, aiming to ensure the AGAN model achieves the best results in the task of cardiac disease diagnosis data imputation.

In order to evaluate the performance of the proposed missing data filling method, a complete dataset without any missing values is used. The missing data is artificially generated by randomly selecting a subset of values from the original dataset and replacing them with the missing marker “NaN” to obtain the incomplete dataset to be filled. The amount of missing data in the incomplete dataset is determined by the missing rate, which is varied between 10% and 60% in this experiment. The complete random missing mechanism is used to ensure that the missing values are randomly distributed and avoid any bias towards specific attributes or samples.

**Fig 7:**
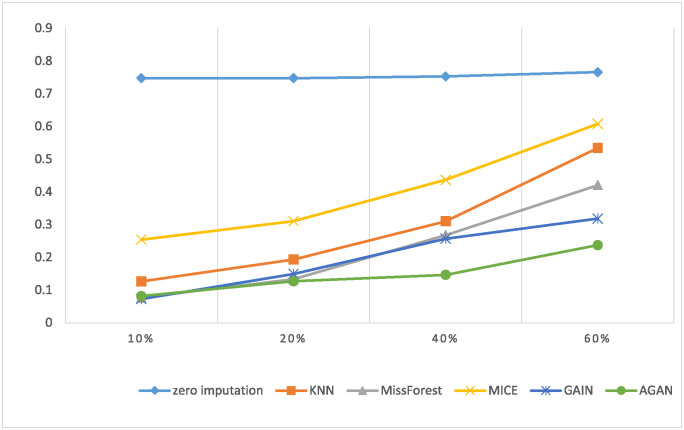
Loss of generator and discriminator during training.

To validate the effectiveness of the model’s imputation, this section conducted 20 independent experimental iterations on two types of cardiac datasets, each iteration utilizing a different random seed to ensure the diversity of the results. Subsequently, we calculated the mean and standard deviation of these repeated experiments to reflect the stability of the model’s performance. Comparative experiments and analyses were carried out using commonly used filling algorithms and models. The experiments included filling tests with different filling methods at various missing rates, and the resulting data were evaluated using RMSE, PFC, and AUC indicators. The differences in experimental results were briefly analyzed. Fig 7 shows the loss of the generator and discriminator in the experiment. In these experiments, various methods are employed to fill the missing heart disease data. The filling methods include zero imputation, missing forest filling [32], multiple imputation by chained equations (MICE), K nearest neighbor(KNN), GAIN model, and AGAN, which is the filling algorithm proposed in this paper. AGAN and GAIN are generative machine learning models, while MissForest, MICE, and KNN are discriminative machine learning models [33]. Zero imputation is used as a reference for comparison.

**Fig 8:**
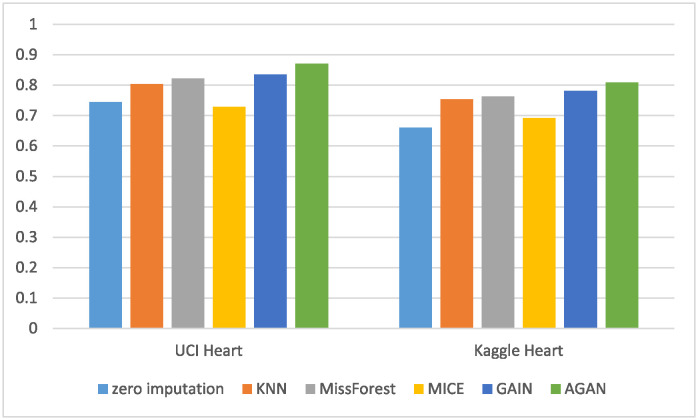
RMSE value of different missing rates on UCI Heart dataset.

**Fig 9:**
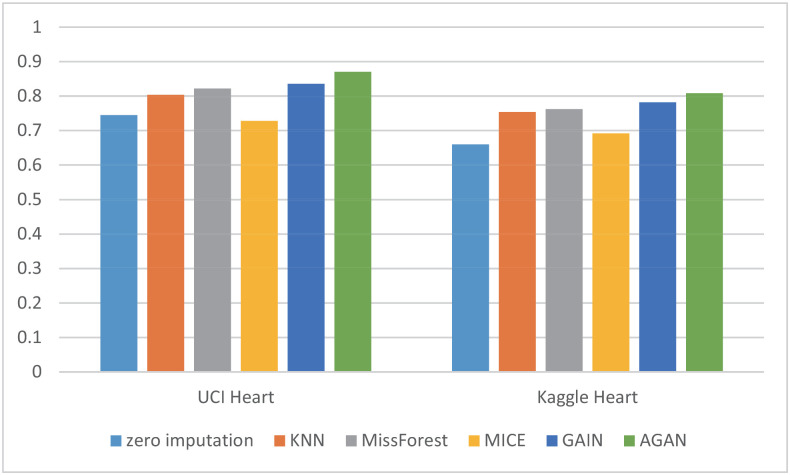
Histogram of classification the AUC indicators for classification after imputing the heart disease dataset.

**Fig 10:**
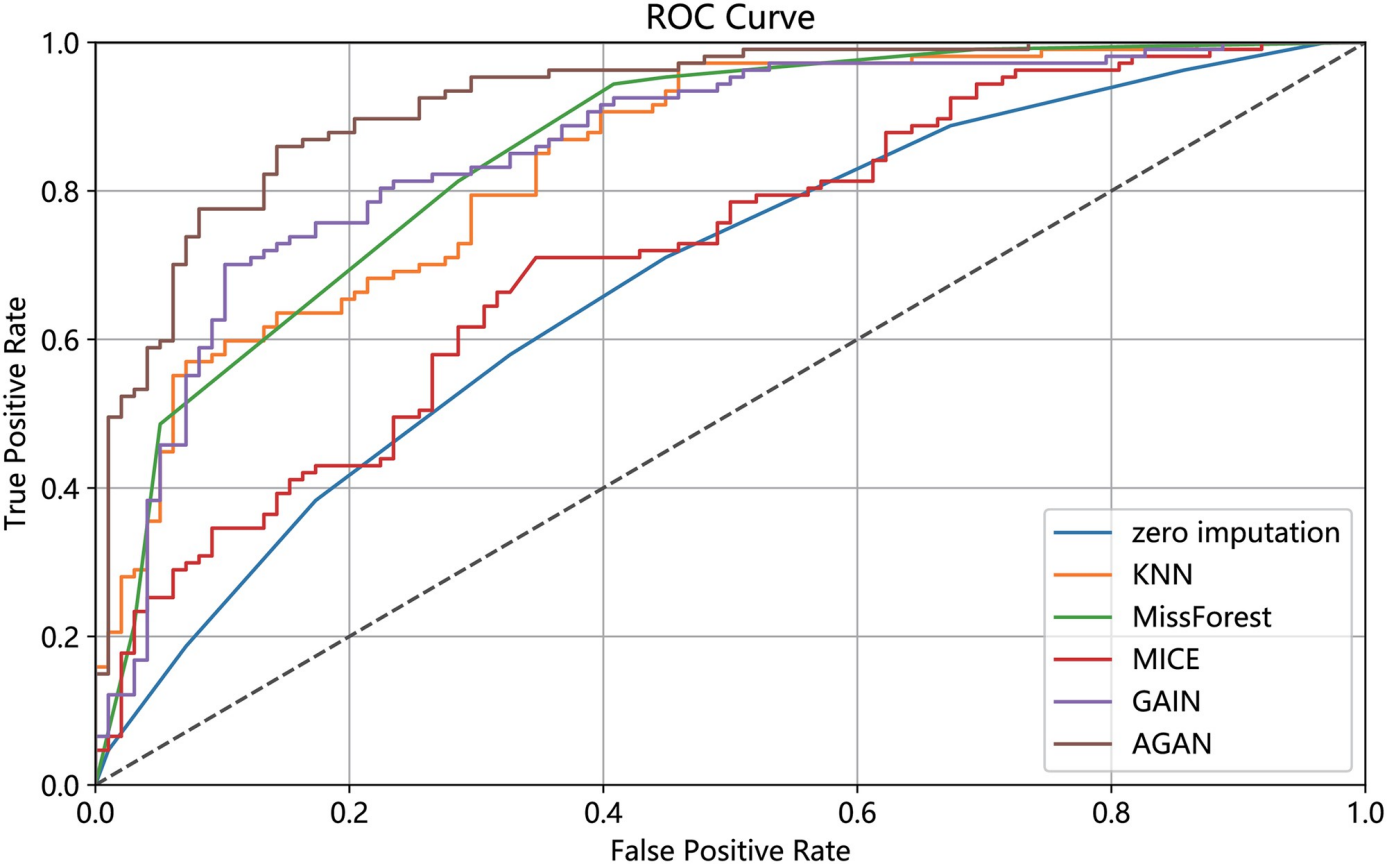
ROC curves of six algorithms.

1. RMSE of missing data filling algorithms on different datasets
  This experiment compares the filling effects of six different methods on two datasets, and Table 4 shows the root mean square error of filling for each method. The experiment utilizes data with a missing rate of 30%. Table 4 demonstrates the notable influence of data dimension and features on the RMSE filled with missing data. Both experimental datasets possess distinct data dimensions and features. In the case of UCI Heart, a dataset with low data dimensions and few attributes, AGAN showcases the best filling effect, with GAIN exhibiting a minor disparity from the discriminant machine learning model. For instance, the filling effect of MissForest slightly surpasses that of GAIN. Conversely, on the dataset Kaggle Heart, which has higher data dimensions and more attributes, AGAN exhibits the smallest error, with an RMSE value of 0.1095, approximately 26% lower than that of the GAIN model and about 30% lower than that of MissForest, which displays the best effect in the discriminant model.
2. RMSE of missing data filling algorithms under different missing rates
  This experiment conducted a comparison of the filling effect of six algorithms on the UCI Heart dataset. The root mean squared error RMSE of the filling is presented in Table 5. The experiment employed data with missing rates ranging from 10% to 60%.
  For ease of display, a visualization diagram depicting the table is presented in Fig 8.
  The visualization diagram in Fig 8 demonstrates that the filled RMSE error gradually increases with the increase of the missing rate. At a 10% deletion rate, AGAN exhibits an RMSE value of only 0.0814. MissForest, GAIN, and AGAN show the smallest filling error at low missing rates. As the missing rate exceeds 40%, the filling effect error of the discriminant algorithm increases significantly, while the algorithm based on the generation of confrontation network shows a slower increase in RMSE value. Compared to GAIN, the AGAN proposed in this study achieves the best filling effect and the smallest error.
3. RMSE of missing data filling algorithms in different sizes
  This experiment compares the filling effect of six algorithms on Kaggle Heart datasets of varying sizes, ranging from 5000 to 30000. Table 6 shows the filled root mean square error and standard deviation. The missing rate of the data used in this experiment is 30%.
  When controlling for the number of features and the missing proportion of control variables, specifically a number of 17 variables and a 30% missing proportion, the result spresented in the table are obtained by calculating four datasets of varying sizes. The table reveals that the RMSE of the four missing values filled by AGAN is the smallest, indicating the best filling effect. GAIN and MissForest follow AGAN in their filling effect, while the MICE algorithm performs the worst under these conditions. When looking at the RMSE value, GAIN’s effect is similar to that of MissForest, while the effect of AGAN is approximately 24% higher than that of GAIN, and approximately 83% higher than that of the reference algorithm.
4. PFC of missing data filling algorithms on different datasets
  This experiment compares the PFC of six methods on UCI Heart and Kaggle Heart datasets. The PFC is shown in Table 7, and the missing rate of the data used in this experiment is 30%. The PFC indicator measures the proportion of missing category variables that are not accurately represented in the reconstructed data. For instance, in the table, the first data point (0.2577) indicates that 25.77% of the missing category variables were filled with an incorrect category using the zero-imputation method. A more precise PFC indicator correlates with a better quality of data reconstruction. It is worth noting that the PFC index for category variables demonstrates that the filling ability of the six imputation algorithms, including zero imputation, MissForest, KNN, MICE, GAIN, and AGAN for missing data, increases in that order. This observation aligns with the results obtained from the evaluation of the RMSE.
5. AUC of downstream classification tasks with 20% missing rate Table 8 presents the AUC values for the downstream classification task with a missing rate of 20%.
  To enhance the visual representation of the results, the data in the table is presented in the form of a histogram, illustrated in Fig 9. Fig 10 displays the ROC curve of the UCI Heart dataset with missing rate of 20% demonstrating the AUC values for downstream classification tasks using different algorithms to fill the complete dataset. A higher AUC value indicates better classification effectiveness. The ROC curve in Fig 10 indicates that the classification effect of the filling algorithm based on generative adversarial networks is superior to that based on discriminative algorithms. Moreover, the AGAN model proposed in this study surpasses previous algorithms in performance.
6. Although the AGAN model, due to its complex structure, requires a longer training time, this does not affect its real-time applicability in cardiac disease diagnosis. This is because model training is a pre-completed, independent phase; once the training is finished and its accuracy verified, the model can be deployed for real-time diagnostics. In practical applications, the key is the model’s inference time, which is the time taken to process new data and provide results, and our tests have shown that the AGAN model is fast and effective in this regard. Furthermore, we are committed to further optimizing the model structure and training algorithms to enhance efficiency. We are also exploring efficient model deployment strategies, such as cloud computing and edge computing technologies, to ensure that the model meets the needs of rapid diagnosis in clinical practice.
7. Methods based on Copula and privacy-preserving approaches, particularly Differential Privacy techniques, have become important research directions in the fields of machine learning and data processing. These methods are especially important when dealing with data that is sensitive to privacy, such as the Differential Privacy technology widely adopted by companies like Google and Apple. This focus reflects the growing importance of protecting individual privacy during data processing and analysis. Our research is primarily focused on improving the accuracy of missing data imputation through Generative Adversarial Networks (GANs). Currently, while our method does not directly integrate Differential Privacy or similar privacy protection mechanisms, we recognize that this is an important direction for future work. In future research, we plan to explore how to integrate privacy protection technologies, such as Differential Privacy, into our framework to protect user privacy while handling sensitive data. It is worth emphasizing that GAN algorithms themselves have great diversity and flexibility. By feeding different noises into the generator, we are able to produce a range of logical and diversified outcomes. This not only enhances the diversity of the model’s generated data but also helps protect patient privacy to some extent. In generating medical data, this approach can avoid revealing specific personal health information while ensuring that the generated data remains statistically useful and relevant. Therefore, combining Differential Privacy technology with this characteristic of GANs holds promise for improving the effectiveness and accuracy of data imputation while protecting privacy.

**Table 4:** RMSE value of missing data filling algorithms on different datasets (mean ± std).

algorithm	UCI Heart	Kaggle Heart
zero imputation	0.7481±.0063	0.5862±.0049
KNN	0.2419±.0050	0.2033±0034
MissForest	0.1637±.0057	0.1580±.0020
MICE	0.2943±.0021	0.2649±.0032
GAIN	0.1752±.0036	0.1481±.0012
AGAN	0.1284±.0011	0.1095±.0028

**Table 5:** RMSE value of missing data filling algorithms on different datasets (mean ± std).

algorithm	10%	20%	40%	60%
zero imputation	0.7474±.00032	0.7472±.0029	0.7527±.0048	0.7657±.0034
KNN	0.1263±.0012	0.1933±.0003	0.3101±.0080	0.5340±.0049
MissForest	0.0768±.0036	0.1329±.0035	0.2676±.0033	0.4205±.0061
MICE	0.2540±.0019	0.3105±.0011	0.4367±.0045	0.6076±.0051
GAIN	0.0727±.0030	0.1491±.0005	0.2569±.0078	0.3181±.0063
AGAN	0.0814±.0021	0.1269±.0014	0.1462±.0026	0.2371±.0057

**Table 6:** RMSE value filled by different algorithms (mean ± std).

algorithm	5000*17	10000*17	20000*17	30000*17
zero imputation	0.7024±.0039	0.6243±.0059	0.6747±.0062	0.6065±.0048
KNN	0.2389±.0012	0.2032±.0003	0.2139±.0080	0.2165±.0037
MissForest	0.1562±.0028	0.1781±.0052	0.1591±.0037	0.1619±.0021
MICE	0.2494±.0010	0.2817±.0031	0.2588±.0037	0.2576±.0029
GAIN	0.1697±.0021	0.1494±.0025	0.1737±.0090	0.1773±.0011
AGAN	0.1250±.0027	0.1241±.0034	0.1585±.0013	0.1363±.0014

**Table 7:** PFC value of algorithms on two datasets.

algorithm	UCI Heart	Kaggle Heart
zero imputation	0.2577	0.2682
KNN	0.1737	0.1773
MissForest	0.1312	0.1427
MICE	0.1854	0.2055
GAIN	0.1230	0.1415
AGAN	0.0912	0.1013

**Table 8:** AUC indicators of classified tasks.

algorithm	UCI Heart	Kaggle Heart
zero imputation	0.7445	0.6598
KNN	0.8042	0.7542
MissForest	0.8221	0.7626
MICE	0.7279	0.6922
GAIN	0.8359	0.7819
AGAN	0.8706	0.8085

## Conclusion

In many domains, including medicine, missing data is a common issue in datasets. In this paper, we propose a algorithm for filling missing data in cardiac disease datasets using generative adversarial networks. Our approach involves introducing an attribute matrix to aid in filling in missing variables, and using the Swish activation function to address gradient disappearance and explosion during training. We conducted six experiments to evaluate the proposed model and analyzed the results. Our findings show that our missing data filling algorithm based on generative adversarial networks outperforms the missing data filling algorithm based on discriminant, as evidenced by significantly better RMSE and AUC. The error rate of our algorithm is over 10% lower than that of the experimental comparison algorithm. Additionally, our approach achieves the lowest RMSE and highest AUC in the experimental comparison algorithm. We also observe that as the missing rate increases, the growth of RMSE is relatively slow. Future research can focus on improving network sparsity algorithm s and optimizing neural network structures based on attribute correlations to enhance interpretability and gap filling. Other potential areas of research include incorporating convolution, BatchNormal, and other technical means to improve network efficiency.

In the paper, the response is as follows: We emphasize that the AGAN model transcends simple missing value replacement strategies, focusing instead on deeply learning and simulating the underlying distribution and features of the data. Despite the large number of zero values (missing values) in the original data, which seemingly simplifies the task for the discriminator, the discriminator actually learns to distinguish between real and generated data based on complex features during the training process, rather than merely the presence of missing values. The goal of the generator is not just to replace missing values, but to generate statistically meaningful fill values based on the intrinsic patterns of the data. Our experimental results confirm that this approach not only improves the accuracy of data imputation but also enhances the relevance and usability of the data, especially in specialized fields like cardiac disease diagnosis. Therefore, AGAN demonstrates significant advantages and potential in handling complex medical data, proving its value and effectiveness in the field of data imputation.
